# Predictors of readmission in hospitalized heart failure patients

**DOI:** 10.34172/jcvtr.2022.08

**Published:** 2022-03-12

**Authors:** Nasim Naderi, Maryam Chenaghlou, Marzieh Mirtajaddini, Zeinab Norouzi, Nasibeh Mohammadi, Ahmad Amin, Sepideh Taghavi, Hamidreza Pasha, Reza Golpira

**Affiliations:** ^1^Rajaie Cardiovascular Medical and Research Center, Iran University of Medical Sciences, Tehran,Iran; ^2^Cardiovascular Research Center, Tabriz University of Medical Sciences, Tabriz, Iran; ^3^Zanjan University of Medical Sciences, Zanjan, Iran

**Keywords:** Heart Failure, Hospitalization, Readmission, Predictors

## Abstract

***Introduction:*** Heart failure(HF) related hospitalization constitutes a significant proportion of healthcare cost. Unchanging rates of readmission during recent years, shows the importance of addressing this problem.

***Methods:*** Patients admitted with heart failure diagnosis in our institution during April 2018to August 2018 were selected. Clinical, para-clinical and imaging data were recorded. All included patients were followed up for 6 months. The primary endpoints of the study were prevalence of early readmission and the predictors of that. Secondary end points were in-hospital and 6-month post-discharge mortality rate and late readmission rate.

***Results:*** After excluding 94 patients due to missing data, 428 patients were selected. Mean age of patients was 58.5 years (±17.4) and 61% of patients were male. During follow-up, 99patients (24%) were readmitted. Early re-admission (30-day) occurred in 27 of the patients(6.6%). The predictors of readmission were older age (*P* = 0.006), lower LVEF (*P* <0.0001), higher body weight (*P* = 0.01), ICD/CRT implantation (*P* = 0.001), Lower sodium (*P* = 0.01), higher Pro-BNP(*P* = 0.01), Higher WBC count (*P* = 0.01) and higher BUN level (*P* = 0.02). Independent predictors of early readmission were history of device implantation (*P* = 0.007), lower LVEF (*P* = 0.016), QRS duration more than 120 ms (*P* = 0.037), higher levels of BUN (*P* = 0.008), higher levels of Pro-BNP(*P* = 0.037) and higher levels of uric acid (*P* = 0.035). Secondary end points including in-hospital and 6-month post-discharge mortality occurred in 11% and 14.4% of patients respectively.

***Conclusion:*** Lower age of our heart failure patients and high prevalence of ischemic cardiomyopathy, necessitate focusing on more preventable factors related to heart failure.

## Introduction

Heart failure (HF) is associated with high morbidity and mortality. The prevalence of HF ranges from 1% and 10% in people under 50 and over 80 years respectively.^1^

The burden of the HF cost of the health care system is high and heart failure is one of the most common reasons of re-hospitalization.^2^

Although the HF admission rate has declined during the past two decades, but this is not true for readmission rate.^3^

Etiologies of HF re-hospitalization are multifactorial, including disease-centered factors, health care-centered factors. Besides, recent changes in medical treatment of HF have affected HF readmission rate.^4^

Despite improvement in the treatment of HF, 30-day and 6-month readmission rates are as high as 20% and 50%. In addition to the above mentioned factors, the health system is probably one of the important related factors of rehospitalization.^5^

Prevalence of HF and also the prevalence of HF risk factors are quite variable across the world. Ischemic heart disease is most prevalent in North America and Europe, whereas valvular heart disease is more common in Asia-Pacific and East Asia.^6^ These diversities necessitate local investigations for better strategy defining. Although there are multiple studies in some regions, there are limited data in our country, Iran.

## Materials and Methods

### Patient selection

In this study, admitted patients with acute heart failure diagnosis in our center between April 2018 to August 2018, were included and registered in Rajaie Acute Heart Failure (RASHF) registry.

### Inclusion criteria:

Patients with acute heart failure reduced ejection fraction (HFrEF) diagnosis based on HF guideline^7^ and older than 16 years were entered in the study.

### Exclusion criteria

Patients with incomplete medical recording data or missing follow up were excluded. We also excluded patients with advanced heart failure who had monthly or bimonthly admission appointment for receiving inotrope and diuretic.

### Data acquisition

Data were derived from Rajaie Acute Systolic Heart Failure Registry (RASHF), the first heart failure registry in Iran. This registry was started in Rajaie Cardiovascular, Medical and research center, Tehran, Iran, a tertiary center of cardiovascular medicine, based on data from hospitalized patients with acute heart failure diagnosis.

The data include the following items, medical history of patients, type of heart failure presentation (denovo or decompensated), cardiomyopathy type (ischemic or non-ischemic), admission-time vital signs, initial clinical symptoms, precipitating factors of acute heart failure, preadmission medications, laboratory findings, baseline ECG and echocardiographic findings, medications during admission and at discharge, in-hospital course and outcome status.

The data were collected from medical records and then entered in the questionnaire. These data were recorded in Regitory system, a software that were designed by the medical Information Technology team of Rajaie Heart Center. The data were collected by a trained general practitioner and the validity of data was controlled by a fellowship of heart failure. The study was approved by the Institutional Research and Ethics committee of our center (Rajaie Heart Center) under the ethics code number of RHC.AC.IR.REC.1396.63. Due to the omitted name of patients, there was no need for writing informed consent. The patients were followed up six months after discharge and re-hospitalized or dead cases were recorded.

### The study end points

The primary endpoints of the study were prevalence of early readmission and the related predictors of it. Secondary endpoints were in-hospital and 6-month post-discharge mortality rate and late readmission rate.

### Statistical analysis

SPSS version 19 was used for statistical analysis. Data are expressed as mean ( ± standard deviation), median (interquartile range) or frequencies as appropriate. Normal distribution of variables was assessed by One-Sample Kolmogorov-Smirnov test. Χ^2^test, Kruskal-Wallistests or Wilcoxon signed rank test, paired t test or Mann-Whitney U-test and students’ t-test, were used for comparisons and associations of variables as appropriate. For determining independent predictors, binary multivariable regression analysis with step-wise selection method was used. *P* value < 0.05 was considered as significant.

## Results

During the study period, data of 522 patients were recorded. After excluding 94 patients based on exclusion criteria, 408 patients were included. Table 1 shows demographic and clinical features of patients. Mean age of patients was 58.59 years. The majority of patients were male (61.3%). The most common type of acute heart failure was chronic decompensated heart failure (93.3%) and 51 % of patients had ischemic cardiomyopathy.

**Table 1:** Demographic and clinical characteristics of patients

**Variable**	**Value**
Age, years (mean ± SD)	58.59 ± 17.44
Male (%)	61.3
Weight, kilogram (mean ± SD)	74.5 ± 18.75
SBP (mean ± SD)	113.51 ± 68
DBP (mean ± SD)	71.21 ± 20.37
HR (mean ± SD)	85.11 22.92
Type of acute heart failure (chronic decompensated) (%)	93.3
Type of heart failure (ischemic) (%)	51
Comorbidities (%)	
Hypertension	42.7
Diabetes	37.5
Dyslipidemia	33.8
CAD	54.9
Peripheral vascular disease	3.7
CKD	35.3
Atrial fibrillation	38
Connective tissue disease	3.7
Cardiac device (ICD/CRT/PPM)	21.9
Smoking	27.5
Drug abuse	2.1
History of chemotherapy/ radiation	4.2
Sign and symptom (%)	
Dyspnea	89.8
Orthopnea	33.8
Paroxysmal nocturnal dyspnea	16.6
GI symptoms	18.6
Abdominal swelling	9.4
Limb swelling	4.7
Increased JVP	15.2
Ascites	17.7
Chest pain	15.5
ECG and echocardiographic findings (%)	
AF rhythm	33.7
Wide QRS	38.7
Severe LV enlargement	35.9
Severe RV enlargement	22.3
More than moderate MR	38.2
More than moderate TR	47.1
Dilated IVC with < 50% collapse	61.2
Pericardial effusion	10
Drug used during hospitalization (%)	
Inotrope	37.4
Infection requiring therapy	49.9
Nitrate	39.5
Metolazone	15
Acetazolamide	7.5

Abbreviations: SBP, systolic blood pressure; DBP, diastolic blood pressure; HR, heart rate; CAD, coronary artery disease; CKD, chronic kidney disease, ICD, implantable cardioverter defibrillator; CRT, cardiac resynchronization therapy; PPM, permanent pacemaker; GI, gasterointestinal; JVP, jugular venous pressure; ECG, electrocardiogram; AF, atrial fibrillation

The most common comorbidity was coronary artery disease (CAD) (54.9%) followed by hypertension (42.7%) and Diabetes (37.5%).

The most prevalent symptom was dyspnea (89.8%) followed by orthopnea (33.8%) and Gasterointestinal (GI) symptoms (18.6%).

Atrial fibrillation (AF) rhythm was detected in 33.7% of patients. The most common echocardiography finding was dilated Inferior Vana Cava (IVC) with reduced collapse (61.2%). Intravenous diuretic was used in 86.8 % and inotrope in 37.4% of patients.

Re-hospitalization occurred in 99 of the patients (24%) and 27 of the patients (6.6%) were readmitted within a month after discharge. The mortality rate was 25.5% and in-hospital death occurred in 45 patients (11%).

Among re-hospitalized patients, 67% of them were admitted in our hospital. Twenty four patients (6%) had ≥ 2 admissions.

The median (IQR) time duration of the index hospitalization to the first readmission was 66 (30-120) days. In patients with more than 2 readmissions within 6 months, the median (IQR) time duration from the first re-hospitalization to second re-hospitalization was 55 (34-101) days.

In univariate analysis, younger age, lower LVEF, higher weight, history of Implantable Cardioverter-Defibrillator/Cardiac resynchronization Therapy (ICD/CRT) implantation, lower sodium level, higher White Blood Cells (WBC), pro B-type Natriuretic Peptide (pro-BNP) and second day Blood Urea Nitrogen (BUN) had correlation with readmission. (Table 2)

**Table 2:** Comparison of variables between groups with and without early readmission

**Variable**	**Early rehospitalization**		***P*** ** value**
	**yes**	**no**	
Age, mean (SD)	49.7(14.3)	59.2(17.5)	0.006
Sex, male, n(%)	20(74.1)	230(60.4)	0.1
LVEF	12.7(7.2)	23(13.6)	< 0.0001
NYHA CLASS	3(3-3.7)	3(3-3.5)	0.1
Weight	84(75-96)	73(62-84)	0.01
SBP	104(85-113)	111(94-130)	0.09
DBP	70(60-87)	70(58-87)	0.5
HF type, HFREF, n, (%)	26(96.3)	331(87)	0.3
HTN	10(40)	156(43)	0.7
DM	6(24)	140(38)	0.1
Smoking	10(40)	97(27)	0.1
CAD	10(42)	187(56)	0.1
CKD	7(28)	129(36)	0.4
AF	9(36)	138(39)	0.7
ICD/CRT	12(48)	72(20)	0.001
TAPSE	12(9-14)	14(12-17)	0.1
RVSm	7(6-10)	7(6-9)	0.8
TRG	47(37-52)	35(25-45)	0.5
More than RV dysfunction	11(52)	128(37)	0.1
QRS > 120	11(48)	135(38)	0.3
Dyspnea	19(90)	316(90)	0.9
BUN	51(36)	39(29)	0.05
Cr	2.1(1.3)	1.7(1.4)	0.1
Na	132(7.5)	135(5.1)	0.01
K	4.4(0.6)	4.3(0.6)	0.1
ALT	30(16-71)	25(18-42)	0.2
AST	38(22-70)	27(19-41)	0.05
ALK	226(184-317)	226(173-340)	0.8
Total bilirubin	1.45(1-2.7)	1.2(0.8-1.9)	0.2
WBC	9350(6959-11625)	7700(6200-10075)	0.01
Uric acid	8.5(2)	7.8(2.8)	0.1
Pro-BNP	12522(6588-20258)	6396(2378-14234)	0.01
Hb	11.8(2.5)	12.1(2.2)	0.4
BUN2	56(37)	43(27)	0.02
Cr2	1.98(1.5)	1.6(0.9)	0.07
Na2	133(7)	135(4.6)	0.007
K	4.2(0.7)	4.2(0.5)	0.9
Total IV diuretic dose	510(247.5-1245)	530(260-1165)	0.9
Length of hospital stay	12(5-17)	9(6-15)	0.3

Abbreviations: LVEF, left ventricular ejection fraction; SBP, systolic blood pressure; DBP, diastolic blood pressure; HF, heart failure; HFrEF, heart failure reduced ejection fraction; HTN, hypertension; DM, diabetes mellitus; CAD, coronary artery disease; CKD, chronic kidney disease; AF, atrial fibrillation; ICD, implantable cardioverter defibrillator, CRT, cardiac resynchronization therapy; TAPSE, tricuspid annular plane systolic excursion; RVsm, right ventricle myocardial systolic velocity; TRG, tricuspid regurgitant gradient, RV, right ventricle, BUN, blood urea nitrogen; Cr, creatinine; Na, sodium; K, potassium; ALT, alanine transaminase; AST, aspartate transaminase; ALK, alkaline phosphatase, Hb, hemoglobin; BUN 2, second day blood urea nitrogen; Cr2, second day creatinine; Na, Second day sodium; IV, intravenous

In multivariate analysis, history of ICD/CRT implantation, lower LVEF, wide QRS, higher BUN, pro-BNP and uric acid level were independent predictors of re-hospitalization. (Table 3)

**Table 3:** Multivariate analysis for determining independent predictors of early re-hospitalization

**Variable**	**OR**	**CI**	***P*** ** value**
Cardiac device (CRT/ICD/PPM)	23.82	2.33-242.59	0.007
LVEF	0.72	0.55-0.94	0.016
Wide QRS	0.13	0.02-0.88	0.037
BUN	1.04	1.01-1.07	0.008
Pro BNP	1.00	1.00-1.00	0.037
Uric acid	0.61	0.39-0.96	0.035

Abbreviations: OR, odds ratio; CI, confidence interval; CRT, cardiac resynchonization therapy; ICD, implantable cardioverter defibrillator; CRT, cardiac resynchronization therapy; PPM, permanent pacemaker; LVEF, left ventricular ejection fraction; BUN, blood urea nitrogen

Total mortality rate, including in-hospital and 6-month mortalities was 25.5% (104 patients). In-hospital mortality occurred in 45 patients (11%).

Correlated factors with mortality in univariate analysis were, lower LVEF and weight, lower systolic blood pressure (SBP) and diastolic blood pressure (DBP), AF rhythm, wide QRS, higher BUN and creatinine, lower sodium, higher potassium, Aspartate Transaminase (AST), total bilirubin, WBC, uric acid, pro-BNP, second day BUN and creatinine, lower hemoglobin and second day sodium, higher total Intravenous (IV) diuretic dose and longer hospital stay. (Table 4)

**Table 4:** Comparison of variables between groups with and without mortality

**Variable**	**Mortality**		***P*** ** value**
	**yes**	**no**	
Age,mean(SD)	59.7(17.9)	58.1(17.3)	0.4
Sex,male,n(%)	69(66.3)	181(59.5)	0.2
LVEF	19(12)	23.5(14)	0.005
Weight	67.5(59-79)	75(65-85)	0.001
SBP	96(85-121)	113(97-132)	< 0.0001
DBP	59(50-75)	71(65-85)	< 0.0001
HTN	36(37.5)	130(44.4)	0.2
DM	35(36.5)	111(37.9)	0.8
Smoking	27(28)	80(27)	0.8
CAD	50(59)	147(54)	0.4
CKD	40(43)	96(33)	0.09
AF	46(48.5)	101(35.2)	0.02
ICD/CRT	24(25)	60(20)	0.3
TAPSE	13(12-15)	14(11-17)	0.1
RVSm	8(6-9)	8(6-9)	0.7
TRG	34(25-45)	35(26-45)	0.6
More than RV dysfunction	40(42)	99(37)	0.3
QRS > 120	46(48)	100(35)	0.03
BUN	50(33.2)	36(28.1)	< 0.0001
Cr	2.1(1.9)	1.6(1.2)	0.002
Na	133(5.7)	135(5)	< 0.0001
K	4.4(0.7)	4.2(0.6)	0.03
ALT	25(20-51)	26(18-46)	0.3
AST	29(21-47)	28(19-44)	0.005
ALK	254(204-347)	224(173-346)	0.1
Total bilirubin	1.3(0.9-2.4)	1.2(0.8-2)	0.001
WBC	9500(4300)	8300(3200)	0.003
Uric acid	8.5(3)	7.6(2.7)	0.01
Pro-BNP	13900(6300-27000)	6200(2500-11600)	< 0.0001
Hb	11.4(2.1)	12.4(2.2)	< 0.001
BUN2	53(32)	40(25)	< 0.0001
Cr2	1.8(1.2)	1.6(0.9)	0.02
Na2	134(6.2)	136(4.3)	0.03
K2	4.2(0.5)	4.2(0.5)	0.6
Total IV diuretic dose	640(320-1740)	560(310-1035)	0.002
Length of hospital stay	11(5-17)	9(6-15)	0.02

Abbreviations: LVEF, left ventricular ejection fraction, SBP, systolic blood pressure, DBP: diastolic blood pressure, HF: heart failure, HFrEF: heart failure reduced ejection fraction, HTN: hypertension, DM: diabetes mellitus, CAD: coronary artery disease, CKD: chronic kidney disease, AF: atrial fibrillation, ICD: : implantable cardioverter defibrillator, CRT: cardiac resynchronization therapy, TAPSE: tricuspid annular plane systolic excursion, RVsm: right ventricle myocardial systolic velocity, TRG: tricuspid regurgitant gradient, RV: right ventricle, BUN: blood urea nitrogen, Cr: Creatinine, Na: Sodium, K: Potassium, ALT: alanine transaminase, AST: aspartate transaminase, ALK: alkaline phosphatase, Hb: hemoglobin, BUN 2: second day blood urea nitrogen, Cr2: second day Creatinine, Na: second day sodium, IV: intravenous

In multivariate analysis, higher pro-BNP and lower hemoglobin were independent predictors of mortality. (Table 5)

**Figure 1:**
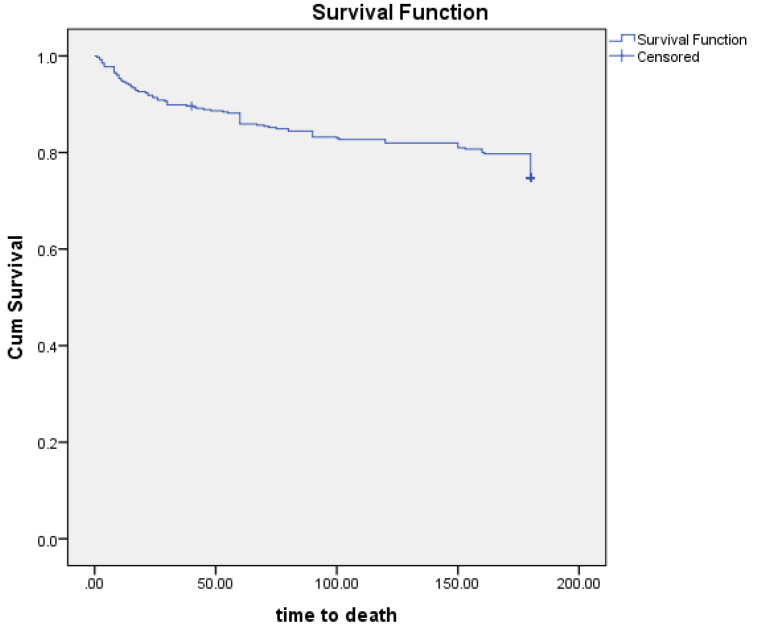
Kaplan Meier curve of survival analysis in study population

Figure 1 shows the Kaplan Meier curve of survival analysis in study population. Time to death is presented in days.

**Table 5:** Multivariate analysis for determining predictors of mortality

**Variable**	**OR**	**CI**	***P*** ** value**
CKD	0.39	0.14-1.08	0.072
Pro BNP	1.00	1.00-1.00	< 0.0001
Hb	0.71	0.57-0.90	0.005

Abbreviations: OR: odds ratio, CI: confidence interval, CKD: chronic kidney disease, Hb: hemoglobin

## Discussion

In the present study, we found several predictors of early readmission in patients with HFrEF enrolled in RASHF registry. The RASHF registry is the first heart failure registry in Iran and the derived data is unique in our country in this regard.

Demographic and clinical features of heart failure have been evaluated in several studies in United States (US) and Europe^8^ but there are limited data about heart failure characteristics in Asia.

In our study, the mean age of our patients was less than 60 years (58.59) and the predominant gender was male (61.35%). Coronary artery disease, hypertension and diabetes were the most common comorbidities respectively. Similar to our results, in a study in Asia, the prevalence of heart failure was higher in men like studies in Europe and US but patients were younger than European studies. Prevalence of comorbidities like hypertension, diabetes and CAD, were similar in some countries of Asia with European and US studies while in others were somewhat different.^9^

In INTER-CHF (International Congestive Heart Failure) study, 5813 patients with heart failure were evaluated in Africa, Asia, Middle East and South America. Mean age of the patients were 53.4, 60, 56.4, 67.1 years, the percentages of male patients were 51.8%, 59.1%, 72.3%, 61.2% and the most common risk factors were as follows, hypertension: 61.6%, 59%, 68.4%, 73.6%, diabetes: 17.1%, 27.9%, 56%, 21.9% and dyslipidemia: 21.1%, 26.1%, 57.1%, 48.7% respectively.^10^

In a study in Africa, the mean age of patients was 56.5 years, 53.6% of patients were female and the three most common etiologies of heart failure were hypertension (45%), rheumatic heart disease (23%) and cardiomyopathy (15%).^11^

It seems the predominant etiology of heart failure affects the demographic features of the disease. For instance, the high prevalence of rheumatic heart disease, lower age of patients and absence of coronary artery disease among the common causes of heart failure in Africa lead to predominance of female gender among the heart failure patients.

Ischemic and hypertensive heart diseases had the highest proportions (26.5% and 26.2%) among the causes of age-standardized HF prevalence in 195 countries and territories from 1990 to 2017.^12^

The rate of re-admission in our study was 24% during 6 months follow-up and of 30-day readmission rate was 6.6%. Despite significant advances in chronic heart failure management, re-hospitalization rate continues to rise and approximately is 30% within 30 to 90 days of discharge ^13^. In a study, the rate of 30-day readmission was 25%^14^ similar to 6-month readmission rate of our study and higher than our 30-day readmission rate.

A study in England, among 698983 HF patients during 2002 to 2018 showed increasing trends of early readmission rate. In this study, readmissions for HF and other cardiovascular diseases remained stable (6% and 3%, respectively) while readmissions for non-cardiovascular diseases increased at a rate of 2.6% per annum.^15^

Numerous models were designed for predicting HF readmission, but their applications in clinical practice were low.

Even a model with application of 26 clinical measures with including nursing assessment data, had fair ability for predicting HF readmission.^16^

A systematic review showed heterogeneous models of re-hospitalized heart failure patients characteristics with significant inconsistencies leading to unavailability to an uniform risk stratification model.^17^

A model based on patient-reported symptoms for assessment of readmission risk has been evaluated, but it seems there is room for progression.^18^

In our study, ICD/CRT implantation, lower LVEF, wide QRS, higher BUN, pro-BNP and uric acid levels were independent predictors of readmission. Somewhat similar findings have been achieved in previous studies.

Some factors have an association with increased risk of readmission. In a study, the presence of chronic renal failure increased the rate of readmission from 26% to 45%.^19^

In another study, clinical factors that were independently have relation with readmission were, chronic obstructive pulmonary disease, systolic heart failure, GFR < 30 ml/min, absence of ACEI/ARB consumption in post discharge period, HIV infection and history of substance abuse. Due to variable predicting risk factors associated with readmission, they concluded that, designing a community specific model is an important strategy for reducing early readmission rates.^20^

Limited studies have been done in this regard in Asia. In a study in Lebanon, history of CAD, diabetes mellitus, gamma glutamyl transpeptidase levels and length of stay were predictors of readmission.^21^

In a large study concluding 28919 patients, factors that have association with readmission after first HF hospitalization were previous hospitalization, age < 65 years, geographic location, length of initial HF hospitalization > 7 days, presence of comorbidities like myocardial infarction, diabetes, stroke and peripheral vascular disease.^22^

In a study in Japan, independent predictors of re-hospitalization were age ≥ 75 years, HR ≥ 75 at discharge, DM and use of loop diuretics at discharge.^23^

In another study, cystatin C, NT pro BNP, cardiac troponin T, diabetes mellitus and NYHA FC III-IV were independent predictors of heart failure readmission and/or mortality. Cystatin C in comparison with conventional kidney function tests was a stronger predictor for the evaluation of adverse events.^24^

In our study, in-hospital and 6-month mortality rates were 11% and 25% respectively.

In-hospital mortality rate ranges from 2.3% in patients entered into clinical trials to 19% in referral center series.^25^

A wide range of in-hospital mortality rate in different studies implies the study population selection effect besides other factors.^26,27,28^

Even after adjusting for differences in patients’ characteristics, HF related factors like post-discharge mortality might have a significant discrepancy between countries.^29^

Our study has been done in a tertiary referral center. The relatively high mortality rate of our study is probably due to this matter.

In our study, independent predictors of mortality were higher Pro BNP and lower hemoglobin.

In concordance with our findings, in a study for determining the profile of hospitalized heart failure patients in a tertiary center, in-hospital mortality rate was 11.2% and some of associated factors of higher mortality were old age, coronary artery disease, atrial fibrillation, renal dysfunction and elevated natriuretic peptide levels.^30^

In a study in Japan, in-hospital mortality rate was 8.3%. Minimum platelet concentration, catecholamine administration, C-reactive protein and total bilirubin levels were predictors of mortality.^31^

Considering all these results reveals that, although there are some similarities between studies regarding various heart failure aspects, but there are significant differences among them and this necessitates local investigations. Based on our finding, it could be recommended that patients with features of high probability of readmission including higher BUN, pro-BNP and uric acid, which are consistent with higher filling pressure and incomplete subsiding hypervolemic state, should be more evaluated for reaching relative euvolemic state.

The main limitation of this study was the missing data. We tried to extract the information from various sources of patients’ documents but cases with frequent missing and unavailable data were omitted. Despite all these arrangements, some missing or incorrect data were unavoidable. The other limitation is the performance of this study in a tertiary center that could not be a surrogate of the all HF patients in our country. Multicenter studies with including different study population could be recommended for future studies.

## Conclusion

Heart failure related admission is one of the most important health care system’s priorities. Different characteristics of this issue all around the world necessitate local specific studies. Relatively younger age of our patients, predominance of ischemic etiology, considerable rate of readmission and in-hospital mortality in this study indicating the importance of addressing this problem.

## Acknowledgements

The authors gratefully acknowledge Hengameh Meschi at the Rajaie cardiovascular, medical and research center for her assistance in this study.

## Funding

None.

## Ethical approval

This study was approved by the ethics committee of Rajaie Cardiovascular Medical and research center under the ethics code number of RHC.AC.IR.REC.1396.63.

## Competing Interests

The authors have no conflicts of interest to declare.
